# Supplementary material for: Identification of photoperiod-induced specific miRNAs in the adrenal glands of Sunite sheep (Ovis aries)
Source: Front Vet Sci. 2022 Jul 22;9:888207. doi: 10.3389/fvets.2022.888207 (PMC9354845; doi:10.3389/fvets.2022.888207)
Supplement: Supplementary file 1 [file Data_Sheet_1.ZIP › Supplementary file/miRDeep2 file.zip!/miRDeep2 file/Know_Structure/SP42A1/oar-mir-362.pdf]

miRBase precursor : oar-mir-362
Total read count : 1177
oar-miR-362 read count : 1165
remaining reads : 12

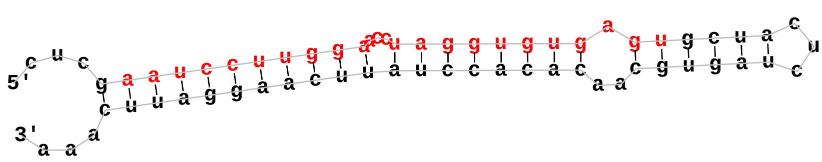

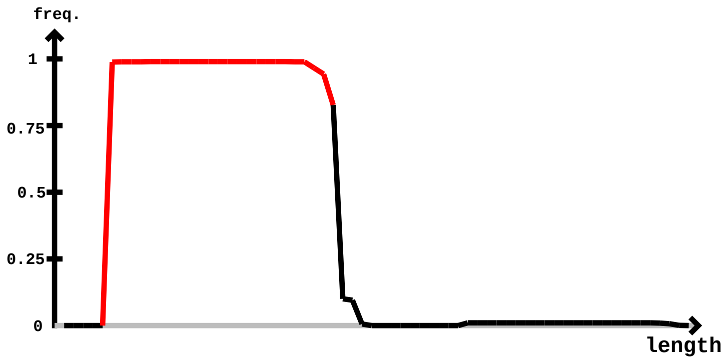


```
        oar-miR-362
5'- cucgaauccuuggaaccuaggugugagugcuacucuagugcaacacaccuauucaaggauucaaa   -3'    exp
    ...(((((((((((...((((((((.((((((...))))))..)))))))))))))))))))...    reads    mm    sample
    ....aauccuuggaaccuaggug..........................................    1        0     seq
    ....aauccuuggaaccuaggugug........................................    26       0     seq
    ....aauccuuggaaccuagguguga.......................................    27       0     seq
    ....aauccuuggaaccuaggugugag......................................    137      0     seq
    ....aauccuuggaaccuaggugugagu.....................................    854      0     seq
    ....aauccuuggaaccuaggugugagug....................................    6        0     seq
    ....aauccuuggaaccuaggugugagugc...................................    105      0     seq
    ....aauccuuggaaccuaggugugagugcu..................................    7        0     seq
    .....auccuuggaaccuaggugugagu.....................................    1        0     seq
    ........cuuggaaccuaggugug........................................    1        0     seq
    .........................................aacacaccuauucaaggauu....    1        0     seq
    .........................................aacacaccuauucaaggauuc...    3        0     seq
    .........................................aacacaccuauucaaggauuca..    7        0     seq
    .........................................aacacaccuauucaaggauucaa.    1        0     seq
```
